# Supplementary material for: Illness (self) management, clinical and functional recovery as determinants of personal recovery in people with severe mental illnesses: A mediation analysis
Source: PLoS One. 2024 Nov 26;19(11):e0313202. doi: 10.1371/journal.pone.0313202 (PMC11594398; doi:10.1371/journal.pone.0313202)
Supplement: S3 Checklist — Checklist of items that should be included in reports of observational studies. (DOCX) [file pone.0313202.s003.docx]

STROBE Statement—checklist of items that should be included in reports of observational studies

|  | | | | | Item No. | Recommendation/ Relevant text from manuscript |  |  | Page  No. |
| --- | --- | --- | --- | --- | --- | --- | --- | --- | --- |
| Title and abstract | | | | | 1 | (***a*) Indicate the study’s design with a commonly used term in the title or the abstract**  Title: Illness (self) management, clinical and functional recovery as determinants of personal recovery in people with severe mental illnesses: A mediation analysis |  |  | 1 |
|  |  |  |  |  |  | **(*b*) Provide in the abstract an informative and balanced summary of what was done and what was found**  Abstract: This study aimed to analyze the association between changes over time in illness self-management skills and personal recovery in patients with schizophrenia and other severe mental illnesses (SMI), and to what degree this association was mediated by changes in clinical and functional recovery. The rationale for the hypothesized directions of association and mediation originated from a recent randomized controlled trial (RCT) on Illness Management and Recovery (IMR), the relations between these concepts suggested in a conceptual framework of IMR, and from the results of three meta-analyses. Moreover, earlier studies indicated the relevance of examining personal recovery as an outcome for people with SMI. Outpatient participants’ data were used in this RCT (N=165). Difference scores were constructed for all concepts by subtracting mean scores measured at baseline (T1) from mean scores at the follow-up measurement (T3). We used mediation analysis to describe pathways between changes in illness management (assessed using the IMR scale client version) and changes in personal recovery (assessed using the Mental-Health Recovery Measure), mediated by changes in clinical (assessed using the Brief Symptom Inventory) and functional recovery (assessed using the Social Functioning Scale). We applied the baseline data of all concepts as covariates. As inferential tests to determine the significance of the indirect paths, confidence intervals were constructed using bootstrap techniques. The results showed that the improvement in overall illness management was directly associated with improvement in personal recovery (B =.32), and indirectly through improvement in clinical recovery (indirect effect =.13) and functional recovery (indirect effect =.09). The main conclusion is that self-reported illness management appears to be more strongly and directly associated with personal recovery than indirectly via clinical and functional recovery. This analysis supports the relevance of self-management interventions such as IMR for the personal recovery of people with SMI. |  |  | 2 |
|  | | |  | Introduction | | | | | |
| Background/ rationale | | | | | 2 | **Explain the scientific background and rationale for the investigation being reported**  Introduction: Patients with serious and persistent mental illnesses (SMIs) such as schizophrenia face major challenges in attaining their personal goals and fully participate in society. This is because of their recurrent symptoms, cognitive limitations, lack of social support, and social impediments such as stigma (1, 2). Psychopharmaceutical treatment can reduce the severity of symptoms and relapse. However, there is also the need for effective psychosocial interventions to provide support for patients in illness self-management. Illness self-management interventions aim to enable people to recover by equipping them with the skills and self-confidence they require to actively recognize and manage their individual health problems (3). Several illness self-management training programs have been developed including Illness Management and Recovery (IMR). The aim of IMR is to achieve progress in recovery by combining better illness management with the pursuit of personal goals (4). IMR is currently used in several countries, such as in the US, European, and Asian countries.  Illness self-management concerns people’s capabilities to take care of themselves and regain control of their lives (5). This includes the ability to perform the tasks needed to manage and live successfully with the physical, social, and emotional consequences of a serious and persistent condition (3). More specifically for people with SMI, it includes the ability to reduce their susceptibility to the illness, and to effectively cope with their symptoms. For example, it is necessary to have knowledge of mental illness to be able to make informed treatment decisions along with professionals, and to have the ability to reach out for social support (5, 6).  Complementary to this, a psychosocial intervention supporting illness self-management includes providing psycho-education and teaching skills for informed decision making on treatment; providing cognitive-behavioral training to support behavioral tailored medication adherence; teaching social and coping skills to deal with symptoms or stress; developing a relapse prevention plan; and improving social support (3, 4).  Recovery is a complex and multi-dimensional concept that has been defined in several ways (7-9). In a typology used in previous studies (10, 11), which will be used in the present study, three types of recovery can be identified, which should be considered not mutually exclusive but complementary aspects of recovery (12)(12). The first type is clinical recovery, which concerns the degree of psychiatric symptomatology (13-15). The second type is functional recovery which can be defined as the degree of vocational and social functioning, such as acting according to age-appropriate role expectations, the performance of daily living tasks without supervision, engagement in social interactions (16), and the degree of independence with regard to housing (9, 17). The third recovery type is personal recovery, a term that emerged from people with a lived experience of mental illness and also emphasizes the personal nature of the recovery process (18, 19). Personal recovery includes several components summarized in the word CHIME (20): connectedness; hope and optimism about the future; identity; meaning in life; and empowerment.  Changes in illness management scores may be related to changes in personal recovery. Previously, a close relationship between illness management and personal recovery was suggested because the improved management of symptoms, relapses, and stresses of everyday life is critical to developing hope and reaching personal recovery goals (5).  IMR is a structured psychosocial program fostering illness self-management in people living with schizophrenia and other SMIs. It was created based on an empirical literature review on teaching strategies for illness self-management (5). The aim of IMR training is to improve illness management skills and thereby improve clinical recovery which improves personal and functional recovery. This working of IMR is suggested in a conceptual framework (4, 21).  Based on this conceptual framework, in a previous study, we cross-sectionally analyzed the association of the components of illness management with the three types of recovery (10). The results showed that one illness management component—coping—was associated with clinical, functional, and personal recovery. The direct associations between coping and functional and personal recovery were stronger than the indirect associations via clinical recovery. One conclusion of this study is that clinical recovery appears to not be a prerequisite for functional and personal recovery (10).  This study was conducted in the context of a randomized controlled trial (RCT) investigating the effects of IMR training + care as usual versus care as usual alone in outpatients with SMI (11, 21). In this RCT we found a significant effect of IMR + care as usual versus care as usual alone in self-reported overall illness management, as well as in personal recovery over an 18 month period (11). Moreover, in this RCT, clinical and functional recovery over time significantly improved about as much in both the IMR training group as in the care as usual group (11).  Based on these results, we wondered whether the found changes in illness self-management might be related to the found changes in personal recovery. If this were the case, we wondered whether this association could be (partially) mediated by changes in clinical and functional recovery.  Rationales for applying those two mediators in the present analysis were: First, in the proposed working of IMR, clinical recovery was a mediator between illness management on the one hand and personal and functional recovery on the other (4). Second, in an earlier review and meta-analysis, illness management appeared to enhance functional recovery (3). Third, in two other earlier reviews and meta-analyses functional recovery was associated with personal recovery (15, 22). Therefore, the research question arose as to whether functional recovery, as well as clinical recovery, might be a mediator that could explain the relationship between illness management and personal recovery.  Therefore, on the basis of the working of IMR suggested in the conceptual framework (4), the results of our RCT (11), and the results of three reviews and meta-analyses, in this study, we were interested to explore to what degree changes in time in overall illness self-management might be associated with changes in personal recovery. Furthermore, we wanted to identify whether these associations might be (partly) mediated by changes in clinical or functional recovery. This research question might be important for future studies to consider which interventions are relevant to improve personal recovery. In this study, mediation analysis was performed to investigate the indirect and direct relations.  Earlier studies have indicated the importance of improvements in personal recovery for people with SMI, including self-perceived growth and leading a satisfactory life despite the presence of persistent symptoms (8, 11, 18, 23, 24). Therefore, examining personal recovery as an outcome of interventions in people with SMI is considered increasingly relevant (15, 22, 25, 26). |  |  | 1-4 |
| Objectives | | | | | 3 | **State specific objectives, including any prespecified hypotheses**  Introduction: This led us to the following research question: What is the association of changes over time in illness self-management skills on changes in personal recovery, and to what degree is this association mediated by changes in clinical and functional recovery?  Statistical analysis: Following our research question, we hypothesized that the improvement of overall illness management had direct and indirect pathways via the improvement in clinical and functional recovery, to the improvement in personal recovery. |  |  | 5  8 |
|  |  | Methods | | | | | | | |
| Study design | | | | | 4 | **Present key elements of study design early in the paper**  Abstract: We used mediation analysis to describe pathways between changes in illness management (assessed using the IMR scale client version) and changes in personal recovery (assessed using the Mental-Health Recovery Measure), mediated by changes in clinical (assessed using the Brief Symptom Inventory) and functional recovery (assessed using the Social Functioning Scale).  Study setting and data collection: For the current analysis, the data on self-rated illness self-management, clinical, functional, and personal recovery were used of 165 (88%) participants in this RCT on IMR of whom both assessments at baseline (T1) and at follow-up (T3) were available, irrespective of treatment condition. The scores on those four domains reflected the status of those participants at those time points. |  |  | 2  7 |
| Setting | | | | | 5 | **Describe the setting, locations, and relevant dates, including periods of recruitment, exposure, follow-up, and data collection**  Study setting and data collection: This analysis involved patients with SMI from two mental health care institutions in the greater Rotterdam area, the Netherlands. Participants were from 14 participating community mental health teams from these institutions. These teams provided rehabilitation-oriented clinical case management.  From Oct. 25, 2012 to May 2, 2014, 187 participants for this RCT were recruited. Randomly (3:2 ratio) 116 participants were assigned to receive IMR + Care as Usual (n = 116) or Care as Usual alone (n = 71). From Oct 5, 2012 to Feb 1, 2017, per study participant assessments were performed at baseline (T1), after 12 months (post-treatment, T2), and after 18 months (six months of follow-up, T3) (11) . |  |  | 6-7 |
| Participants | | | | | 6 | **(*a*) *Mediation analysis* —Give the eligibility criteria, and the sources and methods of selection of participants. Describe methods of follow-up**  Study setting and data collection: This mediation analysis did not require extra assessments of patients as the data from our RCT on IMR were used. Inclusion criteria for this RCT were as follows: age 18–65 years; diagnosis with an SMI such as schizophrenia or a persistent mood disorder with or without comorbid disorders (i.e., substance abuse and personality disorders); receiving outpatient treatment; and being willing and able to give written informed consent. Exclusion criteria comprised previous participation in IMR training and insufficient Dutch language skills. From Oct. 25, 2012 to May 2, 2014, 187 participants for this RCT were recruited through clinician referrals for IMR. These selected clients indicated both a willingness to participate in IMR and to be informed about the study. An assistant researcher explained the study objectives and procedures. If participants agreed to participate in the study, written informed consent was obtained. Randomly (3:2 ratio) 116 participants were assigned to receive IMR + Care as Usual (n = 116) or Care as Usual alone (n = 71).  For the current mediation analysis, the data on self-rated illness self-management, clinical, functional, and personal recovery were used of 165 (88%) participants in this RCT on IMR of whom both assessments at baseline (T1) and at follow-up (T3) were available, irrespective of treatment condition. |  |  | 6-7  7 |
|  |  |  |  |  |  | (*b*) ***Mediation analysis*** —**For matched studies, give matching criteria and number of exposed and unexposed**  Study setting and data collection: For the initial RCT randomly (3:2 ratio) 116 participants were assigned to receive IMR + Care as Usual (n = 116) or Care as Usual alone (n = 71).  For the current mediation analysis, the data on self-rated illness self-management, clinical, functional, and personal recovery were used of 165 (88%) participants in this RCT on IMR of whom both assessments at baseline (T1) and at follow-up (T3) were available, irrespective of treatment condition. Of the 165 participants in this mediation analysis, 104 (63%) in the original RCT belonged to the experimental condition (exposed to care as usual + IMR), and 61 (37%) belonged to the control group (exposed to care as usual, but unexposed to IMR). |  |  | 6-7 |

| Variables | | | 7 | | | **Clearly define all outcomes, exposures, predictors, potential confounders, and effect modifiers. Give diagnostic criteria, if applicable**  Measures: In this mediation analysis, pathways were described between the improvement in illness (self-) management and the improvement in personal recovery, mediated by the improvement in clinical and functional recovery. Therefore self-rated illness self-management, clinical, functional, and personal recovery were all outcome measures. Self-rated illness self-management, clinical, and functional recovery were predictors of personal recovery (outcome). Clinical, and functional recovery were mediators between the relationship of illness self-management and personal recovery. Illness (self-) management (IM) was assessed with the IMR scale client version. Personal recovery (PR) was assessed with the Mental-Health Recovery Measure (MHRM). Clinical recovery (CR) was assessed with the Brief Symptom Inventory (BSI). Functional recovery (FR) was assessed with the Social Functioning Scale (SF-scale).  To avoid regression to the mean, we applied the baseline data of all concepts as covariates. This corrected the correlation between the difference score and the baseline measurement. | | |  |  | | 7-9 | | |  |  |
| --- | --- | --- | --- | --- | --- | --- | --- | --- | --- | --- | --- | --- | --- | --- | --- | --- |
| Data sources/ measurement | | | 8* | | | **For each variable of interest, give sources of data and details of methods of assessment (measurement). Describe comparability of assessment methods if there is more than one group**  Study setting and data collection: This analysis did not require extra assessments of patients as the data from our RCT on IMR (11, 21) were used. Participants for this RCT were recruited through clinician referrals for IMR. These selected clients indicated both a willingness to participate in IMR and to be informed about the study. An assistant researcher explained the study objectives and procedures. If participants agreed to participate in the study, written informed consent was obtained. All four outcome measures used in the mediation analysis were self-rated. As the assessors were blinded to group assignment, the data collection procedure was single-blinded. Assessments were performed at baseline (T1), after 12 months (post-treatment, T2), and after 18 months (six months of follow-up, T3). The research assistants, who were centrally instructed, met the study participants at several institute branches to conduct the assessments. Assessment methods of the original experimental and control group of the RCT were identical. | | |  |  | | 6-7 | | |  |  |
| Bias | | | 9 | | | **Describe any efforts to address potential sources of bias**  In the mediation analysis: Statistical analysis: To avoid regression to the mean, we applied the baseline data of all concepts as covariates. This corrected the correlation between the difference score and the baseline measurement. To facilitate comparability, standardized regression coefficients were used.  In the RCT: As the assessors were blinded to group assignment, the data collection procedure was single-blinded (11). | | |  |  | | 9  7 | | |  |  |
| Study size | |  | 10 **Explain how the study size was arrived at**  In the original RCT: 187 participants for this RCT were recruited through clinician referrals for IMR. We had calculated that this study size  was appropriate for this RCT (11). (Based on the effect sizes observed in the three RCTs on IMR published at the time of designing the RCT,  we anticipated a moderate effect size of 0.40 with respect to the primary outcome variable (i.e., the self-rated IMR scale). Based on power  analyses with three measurement times (mixed models), equal allocation to the experimental and control group a power of 0.80, alpha set at  0.05, and an effect size of 0.40, we determined that it would be necessary to randomize 148 clients: 74 to the experimental condition and 74 to  the control group. Due to the planned 3:2 randomization we aimed for 111 participants (74 x 3:2) in the experimental condition. However, we  succeeded to include 187 participants. They were randomly assigned (3:2 ratio) to receive IMR + CAU (n = 116) or CAU alone (n = 71) (11).  In the mediation analysis: Study setting and data collection: For the current mediation analysis, the data on self-rated illness self-management,  clinical, functional, and personal recovery were used of 165 (88%) participants in this RCT on IMR of whom both assessments at baseline  (T1) and at follow-up (T3) were available, irrespective of treatment condition. | | | | | |  |  | | 6  7 | | |  |  |
| Quantitative variables | | | 11 | | **Explain how quantitative variables were handled in the analyses. If applicable, describe which groupings were chosen and why**  Statistical analysis: In this mediation analysis, pathways were described between the improvement in illness (self-) management (ΔIM) and the improvement in personal recovery (ΔPR), mediated by the improvement in clinical (ΔCR) and functional recovery (ΔFR) (Fig 1). Difference scores were constructed for all concepts by subtracting mean scores measured at T1 from mean scores measured at T3 (38). To avoid regression to the mean, we applied the baseline data of all concepts as covariates (38-40). This corrected the correlation between the difference score and the baseline measurement. The regression-equation for the ΔCR mediator consisted of an intercept, ΔIM, baseline IM, and baseline CR; for the ΔFR mediator the regression-equation consisted of an intercept, ΔIM, baseline IM, and baseline FR; for the ΔPR the regression-equation consisted of an intercept, ΔIM, ΔCR, ΔFR, baseline IM, baseline CR, baseline FR, and baseline PR. To facilitate comparability, standardized regression coefficients were used. The direct path consisted of a regression equation from ΔIM to ΔPR (*c’)*. The two indirect paths consisted of regression equations from ΔIM to the mediators ΔCR (*a1*) and ΔFR (*a2*) respectively, and regression equations from those mediators to ΔPR (corrected for ΔIM to ΔPR) (*b1* and *b2*) (Fig 1). As inferential tests to determine the significance of the indirect paths, confidence intervals were constructed by multiplying the regression weights of ΔIM in the first regression and of the mediators in the second. This was executed on 5000 bootstrap samples of our dataset, applying bias correction. For this purpose, the PROCESS macro was used in SPSS 27. In this type of mediation analysis, using the PROCESS macro, there can be only one predictor and only one outcome, but multiple mediators can be specified (37, 38). | | | |  |  | | 8-9 | | | | |
| Statistical methods | | | 12 | | **(*a*) Describe all statistical methods, including those used to control for confounding**  Statistical analysis: In this mediation analysis, pathways were described between the improvement in illness (self-) management (ΔIM) and the improvement in personal recovery (ΔPR), mediated by the improvement in clinical (ΔCR) and functional recovery (ΔFR) (Fig 1). Difference scores were constructed for all concepts by subtracting mean scores measured at T1 from mean scores measured at T3 (38). To avoid regression to the mean, we applied the baseline data of all concepts as covariates. This corrected the correlation between the difference score and the baseline measurement. The regression-equation for the ΔCR mediator consisted of an intercept, ΔIM, baseline IM, and baseline CR; for the ΔFR mediator the regression-equation consisted of an intercept, ΔIM, baseline IM, and baseline FR; for the ΔPR the regression-equation consisted of an intercept, ΔIM, ΔCR, ΔFR, baseline IM, baseline CR, baseline FR, and baseline PR. To facilitate comparability, standardized regression coefficients were used. The direct path consisted of a regression equation from ΔIM to ΔPR (*c’)*. The two indirect paths consisted of regression equations from ΔIM to the mediators ΔCR (*a1*) and ΔFR (*a2*) respectively, and regression equations from those mediators to ΔPR (corrected for ΔIM to ΔPR) (*b1* and *b2*) (Fig 1). As inferential tests to determine the significance of the indirect paths, confidence intervals were constructed by multiplying the regression weights of ΔIM in the first regression and of the mediators in the second. This was executed on 5000 bootstrap samples of our dataset, applying bias correction. For this purpose, the PROCESS macro was used in SPSS 27. In this type of mediation analysis, using the PROCESS macro, there can be only one predictor and only one outcome, but multiple mediators can be specified (37, 38). | | | |  |  | | 8-9 | | | | |
|  |  |  |  |  | **(*b*) Describe any methods used to examine subgroups and interactions** N/A | | | |  |  | |  | | | | |
|  |  |  |  |  | **(*c*) Explain how missing data were addressed** N/A | | | |  |  | |  | | | | |
|  |  |  |  |  | (*e***) Describe any sensitivity analyses** N/A | | | |  |  | |  | | | | |
|  | Results | | | | | | | | | | | | | | |  |
| Participants | | | 13* | | (a) **Report numbers of individuals at each stage of study—eg numbers potentially eligible, examined for eligibility, confirmed eligible, included in the study, completing follow-up, and analysed.**  For the current mediation analysis, the data on self-rated illness self-management, clinical, functional, and personal recovery were used of 165 (88%) participants in this RCT on IMR of whom both assessments at baseline (T1) and at follow-up (T3) were available, irrespective of treatment condition.  Of the 165 participants in this mediation analysis, 104 (63%) in the original RCT belonged to the experimental condition (care as usual + IMR), and 61 (37%) belonged to the control group (care as usual alone) (Table 1). | | | |  |  | | 7 | | | | |
|  |  |  |  |  | (b) **Give reasons for non-participation at each stage** (see 13a) | | | |  |  | | 7 | | | | |
|  |  |  |  |  | (c) **Consider use of a flow diagram** A flow-diagram of the original RCT is available in our effect-paper (11) | | | |  |  | |  | | | | |
| Descriptive data | | | 14* | | (a) **Give characteristics of study participants (eg demographic, clinical, social) and information on exposures and potential confounders**  The participants were mostly men who lived alone, had a secondary level of education, were born in the Netherlands, and had a psychotic disorder. The modal treatment length was over five years: they had been admitted at least three times, hospitalized for less than one year, and were receiving income from unemployment, disability, or sick leave. Of the 165 participants in this mediation analysis, 104 (63%) in the original RCT belonged to the experimental condition (exposed to care as usual + IMR), and 61 (37%) belonged to the control group (exposed to care as usual, unexposed to IMR)(Table 1). | | | |  |  | | 10-12 | | | | |
|  |  |  |  |  | **(b) Indicate number of participants with missing data for each variable of interest** N/A | | | |  |  | |  | | | | |
| Outcome data | | | 15* | | **Report numbers of outcome events or summary measures**  The original RCT included 187 participants. Per study participant assessments were performed at baseline (T1), after 12 months (post-treatment, T2), and after 18 months (six months of follow-up, T3). For the current mediation analysis, the data on self-rated illness self-management, clinical, functional, and personal recovery were used of 165 (88%) participants in this RCT on IMR of whom both assessments at baseline (T1) and at follow-up (T3) were available, irrespective of treatment condition. | | | |  |  | | 7 | | | | |
| Main results | | | 16 | | (*a*) **Give unadjusted estimates and, if applicable, confounder-adjusted estimates and their precision (eg, 95% confidence interval). Make clear which confounders were adjusted for and why they were included**  From a multiple mediation analysis, it appeared that the improvement in overall illness self-management indirectly affected the improvement of overall personal recovery through its effect on the improvement of clinical recovery and functional recovery. As shown in Fig 2 and Table 2, clients who improved more in overall illness management improved more in clinical recovery and in functional recovery than those clients who improved less in overall illness management, and clients who improved more in clinical recovery or functional recovery also improved more in personal recovery. Both 95% confidence intervals: of the indirect pathways via improvement of clinical recovery (path *a_1_b_1_* = 0.48 x 0.26 = 0.13) and of the pathways via improvement of functional recovery (*a_2_b_2_* = 0.41 x 0.21 = 0.09) were above zero, implying statistical significance. However, it was also shown that improvement in overall illness self-management strongly affected improvement in overall personal recovery independent of its impact on improvement in clinical recovery and its impact on improvement in functional recovery (c' = 0.32) . Therefore, illness self-management appeared to be a stronger direct determinant of personal recovery than indirectly via clinical and functional recovery.  To facilitate comparability all outcomes and predictors were standardized. a1, a2, b1, b2 and c' correspond with Fig 1. a x b, bootstrap results for indirect effects; lower and upper limits of bias corrected confidence intervals for test of mediation with 5,000 bootstrap samples. All scores irrespective of treatment condition, and after correction with baseline data of all variables as covariates. This corrected the correlation between the difference score and the baseline measurement. | | | |  |  | | 12-14  9, 14 | | | | |
|  |  |  |  |  | **(*b*) Report category boundaries when continuous variables were categorized** N/A | | | |  |  | |  | | | | |
|  |  |  |  |  | **(*c*) If relevant, consider translating estimates of relative risk into absolute risk for a meaningful time period** N/A | | | |  |  | |  | | | | |
| Other analyses | | | | 17 | **Report other analyses done—eg analyses of subgroups and interactions, and sensitivity analyses** N/A | | |  |  | |  | |  |  |  |  |
|  | Discussion | | | | | | | | | | | |  |  |  |  |
| Key results | | | | 18 | **Summarise key results with reference to study objectives**  The research question of this study was: What is the association of changes over time in the illness self-management skills of people with SMI on changes in personal recovery, and to what degree is this association mediated by changes in clinical and functional recovery  From a multiple mediation analysis, it appeared that the improvement in overall illness self-management indirectly affected the improvement of overall personal recovery through its effect on the improvement of clinical recovery and functional recovery. As shown in Fig 2 and Table 2, clients who improved more in overall illness management improved more in clinical recovery and in functional recovery than those clients who improved less in overall illness management, and clients who improved more in clinical recovery or functional recovery also improved more in personal recovery. Both confidence intervals: of the indirect pathways via improvement of clinical recovery (path *a_1_b_1_* = 0.48 x 0.26 = 0.13) and of the pathways via improvement of functional recovery (*a_2_b_2_* = 0.41 x 0.21 = 0.09) were above zero, implying statistical significance. However, it was also shown that improvement in overall illness self-management strongly affected improvement in overall personal recovery independent of its impact on improvement in clinical recovery and its impact on improvement in functional recovery (c' = 0.32). Therefore, illness self-management appeared to be a stronger direct determinant of personal recovery than indirectly via clinical and functional recovery. | | |  |  | | 12-14 | |  |  |  |  |
| Limitations | | | | 19 | **Discuss limitations of the study, taking into account sources of potential bias or imprecision. Discuss both direction and magnitude of any potential bias**  This mediation analysis was based on difference scores of all four concepts on two time points; this study, therefore, used longitudinal information, but we were not able to infer causality. This would require more measurement points of time than those in this study. Future research should include longitudinal analysis.  Another limitation is that all outcomes used were self-report questionnaires, which are inherently subjective. This may have caused bias in the associations between the measured concepts. Therefore, future research could also use clinician-rated outcomes, such as the clinician-rated IMR-Scale measuring illness self-management (29), the Brief Psychiatric Rating Scale for clinical recovery (43), and the Social and Occupational Functioning Assessment Scale for functional recovery (44).  Personal recovery should be self-reported. In the present study, the MHRM was used, which is a composite personal recovery scale. However, as there are various measures for personal recovery (22) future research could use another scale for measuring personal recovery, such as the Recovery Assessment Scale (45).. | | |  |  | | 16-17 | |  |  |  |  |
| Interpretation | | | | 20 | **Give a cautious overall interpretation of results considering objectives, limitations, multiplicity of analyses, results from similar studies, and other relevant evidence**  This study’s results showed empirical support for the association between illness self-management and personal recovery. Moreover, self-reported illness management appeared to be a stronger direct determinant of personal recovery than indirectly via clinical and functional recovery. Furthermore, this study confirms that improvement in clinical and functional recovery might not be a prerequisite for improvement in personal recovery**.** Therefore, this study supports the relevance of self-management interventions such as IMR. These mental health services deserve more attention and should be part of the standard mental health care for people with SMI.  The results partly provide empirical support for the working of IMR, as suggested in the conceptual framework. However, in this study, a possible new variant of this IMR framework was demonstrated, because functional recovery could be a mediator between illness management and personal recovery.  Strengths of this study are: (1) This mediation analysis has a firm base (37) because the hypothesized directions of associations  between the concepts examined were derived from theory (4), from previous research results by other researchers (3, 15, 22), and from ourselves (11). This might support the relevance of the present outcomes (37). Moreover, in this study, a possible different working of IMR has been suggested than that previously proposed. This concerns introducing functional recovery as a possible mediator between illness management and personal recovery.  (2) Two meta-analyses respectively investigated the impact of clinical and functional recovery on personal recovery, but not the impact of illness management (15, 22). A separate meta-analysis examined the impact of illness self-management (3). However, in this study, both the indirect role of clinical and functional recovery and the direct role of illness management as determinants of personal recovery were investigated.  This study supports the relevance of self-management interventions such as IMR, which should be part of standard care for people with SMI and should be given priority in guidelines (3).  Considering the limitations of this study, to further investigate our results, future research using a longitudinal analysis and other outcome-measures for measuring the same concepts is recommended. | | | |  |  | | 16-18 | |  |  |  |
| Generalizability | | | | 21 | **Discuss the generalizability (external validity) of the study results**  Our RCT on IMR, from which the data of this mediation analysis are derived, was conducted in a natural setting (11). Therefore, we suggest that the results of the current analysis may be well generalizable. | | |  |  | | 16 | |  |  |  |  |
| Other information | | | | |  | |  | | | | | |  |  |  |  |
| Funding | | | | 22 | **Give the source of funding and the role of the funders for the present study and, if applicable, for the original study on which the present article is based**  Most of the study was funded by Parnassia Psychiatric Institute (The Hague/ Rotterdam). The remainder was funded by an unconditional educational grant from Janssen-Cilag B.V. (1-4H1ZDZ67890). The role of funding source was: none. | | |  |  | | 19 | |  |  |  |  |

*Give information separately for cases and controls in case-control studies and, if applicable, for exposed and unexposed groups in cohort and cross-sectional studies.

**Note:** An Explanation and Elaboration article discusses each checklist item and gives methodological background and published examples of transparent reporting. The STROBE checklist is best used in conjunction with this article (freely available on the Web sites of PLoS Medicine at http://www.plosmedicine.org/, Annals of Internal Medicine at http://www.annals.org/, and Epidemiology at http://www.epidem.com/). Information on the STROBE Initiative is available at www.strobe-statement.org.
