# Supplementary figures and images for: Illness (self) management, clinical and functional recovery as determinants of personal recovery in people with severe mental illnesses: A mediation analysis
Source: PLoS One. 2024 Nov 26;19(11):e0313202. doi: 10.1371/journal.pone.0313202 (PMC11594398; doi:10.1371/journal.pone.0313202)

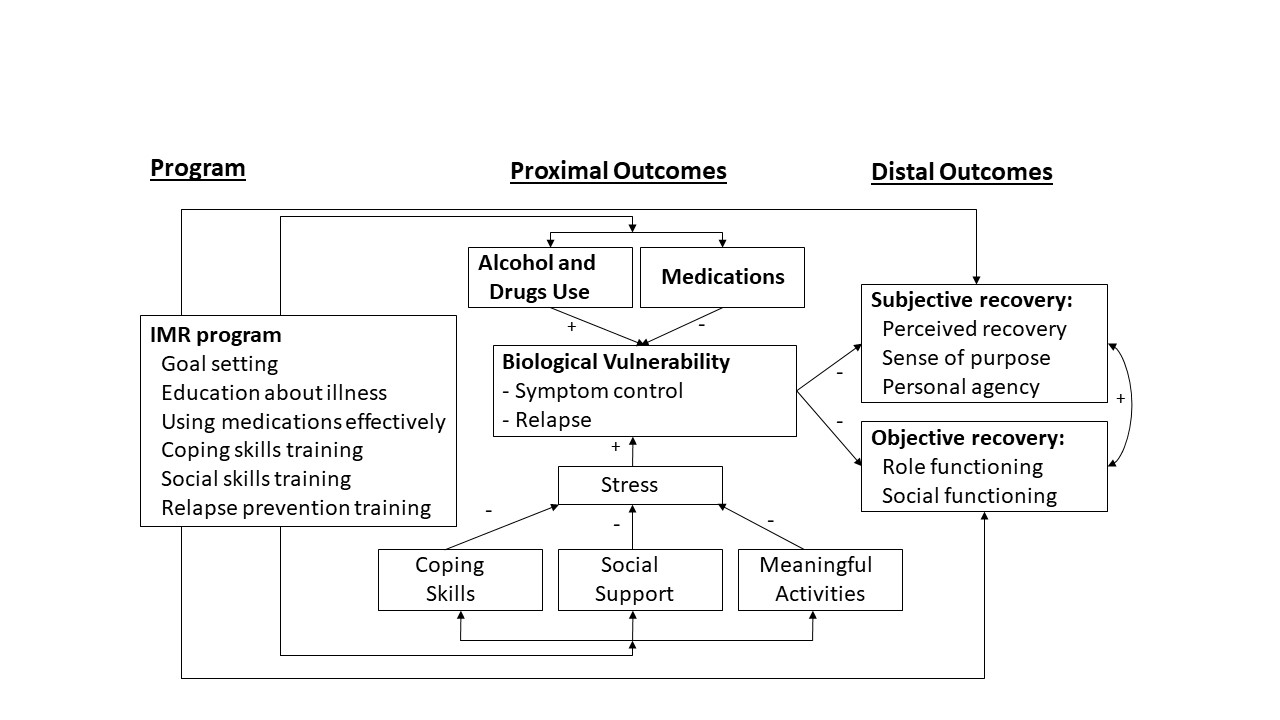

Supplement: S1 Fig — (JPG) [file pone.0313202.s004.JPG]
