# Supplementary material for: Illness (self) management, clinical and functional recovery as determinants of personal recovery in people with severe mental illnesses: A mediation analysis
Source: PLoS One. 2024 Nov 26;19(11):e0313202. doi: 10.1371/journal.pone.0313202 (PMC11594398; doi:10.1371/journal.pone.0313202)
Supplement: Attachment — Submitted filename: Recommendations.pdf [file pone.0313202.s005.pdf]

Recommendations

Illness (self) management, clinical and functional recovery as determinants of personal recovery in people with severe mental illnesses: A mediation analysis

I commend the authors for their thorough and rigorous approach to examining the association between illness self-management skills and personal recovery in patients with SMI. The study's foundation on recent RCTs, meta-analyses, and a conceptual framework lends significant credibility and relevance to the research. The methodologies adopted, especially the use of bootstrap techniques for inferential tests and mediation analysis, reflect a deep understanding and careful consideration of the research question at hand. However, I would like to give suggestions in areas where there's room for improvement as follows.

Abstract

It's not immediately evident why the "clinical trial registration number" is relevant in the abstract. If it doesn't add critical value or context for the reader, consider moving such details to the methods section of the full paper.

Introduction

1. To strengthen the manuscript's foundation, a brief but comprehensive summary of the previous study's methodology and findings would be helpful. This can ensure that readers understand the context in which the previous conclusions were drawn. For each cited study or meta-analysis, it could be beneficial to provide a brief summary of their methods, sample sizes, and key findings to lend weight to the argument.

2. If the prior study was cross-sectional, explain why an RCT is now appropriate and how it advances understanding. RCTs can demonstrate causality, while cross-sectional studies cannot.

3. Beyond the prior study, is there other theoretical or empirical evidence that suggests the possibility of mediating relationships? If so, this should be included to bolster the rationale.

4. It might be beneficial to provide a bit more detail on how and why clinical and functional recovery might mediate the relationship

Method

The manuscript mentions that participants were randomized in a 3:2 ratio to receive either IMR + Care as Usual (CAU) or just Care as Usual (CAU) alone. The actual numbers given are 116 participants for IMR + CAU and 71 participants for CAU alone.

However, there appears to be a discrepancy in the numbers provided based on the mentioned ratio: If you were to split 187 participants (the total mentioned) using a 3:2 ratio: So, if you strictly followed a 3:2 ratio, you'd expect 112 participants in the IMR + CAU group and 75 participants in the CAU alone group. The manuscript provides the numbers as 116 for IMR + CAU and 71 for CAU alone, which is close but not an exact 3:2 ratio. However, in practical applications, especially with human participants, achieving an exact ratio can sometimes be challenging due to various logistical and operational reasons. It's essential to ensure that randomization is properly carried out, regardless of minor discrepancies in the intended ratio. Thus, the authors should add more detail about randomization and why this research can not reach the expected participants.

Measures

1. In the section detailing the research instruments, I strongly recommend providing more information regarding the reliability and validity of each tool used. This will help readers ascertain the robustness of the study's methodology. Furthermore, given the context, it would be pertinent to know if these tools were translated from English to Dutch. If they were, kindly outline the translation process and any cultural adaptations made. Such details ensure the translated tools maintain their original intent and measure the intended constructs accurately

2. I recommend elaborating on the IMR program in the measures section. Given that the IMR is central to the study's objectives, it would provide valuable context for readers who are not deeply familiar with its specifics. A brief overview of the IMR program's key components, objectives, and methodologies will enhance the validity and interpretability of your findings. Furthermore, for the sake of reproducibility and facilitating comparative analysis with other studies, having a clear understanding of the IMR program and its application in this research is essential."

Result

The results seem to rely heavily on different scores (like ΔIM, ΔCR). Discuss the choice to use these scores more thoroughly. Are there any potential pitfalls or benefits in using difference scores as opposed to raw post-intervention scores?

Discussion

1. The statement, "These results suggest that improvement in clinical and functional recovery might not be a prerequisite for improvement in personal recovery," is strong. To solidify this claim, you could provide more supporting evidence from the study and cite any corroborative external studies.

2. You've pointed out that both clinical and functional recovery were mediators, but their effects were 'weak.' This might warrant further exploration. Why were these relationships weak? Were the measures used for clinical and functional recovery robust? Or is it possible that other unmeasured variables play a more significant role?

3. While there's mention of prior studies that align with or differ from the current results, it would be beneficial to discuss more explicitly why these similarities or differences might exist.

4. The discussion concludes with a statement on the relevance of IMR, suggesting it should be standard care. However, the manuscript should expand on why the current findings specifically support this, especially given the noted weak relationships between certain variables.

5. You mention the results of previous meta-analyses to support the findings. Elaborate on how closely these studies align with your study in terms of methodology and findings. Discussing the quality and potential biases of these meta-analyses might also.

6. The manuscript suggests that clinical recovery might not be a prerequisite for personal recovery. However, it also notes that "better symptom management is required to develop hope for the future." This seems contradictory, and the discussion should address such apparent inconsistencies.
