# Supplementary material for: Illness (self) management, clinical and functional recovery as determinants of personal recovery in people with severe mental illnesses: A mediation analysis
Source: PLoS One. 2024 Nov 26;19(11):e0313202. doi: 10.1371/journal.pone.0313202 (PMC11594398; doi:10.1371/journal.pone.0313202)
Supplement: Attachment — Submitted filename: Response to reviewers 10-07-24 .doc [file pone.0313202.s006.doc]

**Response to reviewers**

**Answers on your statements in:** PLOS ONE Decision: Revision required [PONE-D-23-14969], dated 7 Sept 2023

We thank the editors and reviewers for their compliments, constructive remarks, and suggestions. Below, we address all the issues concerning the article for publication in PLOS-ONE PONE-D-23-14969, ‘Illness (self) management, clinical and functional recovery as determinants of personal recovery in people with severe mental illnesses: A mediation analysis.’

All references to line numbers in this response letter refer to the numbers in the present submission.

Reviewer #1:

**1. First, it is not clear why the researchers did not assess change in the constructs of interest in longitudinal models given that there were three data collection time points. This would be a vast improvement over using change scores given problems with reliability and interpretability of change across only two time points.**

**Answer**:

Thank you for your comments. We have amended the first limitation of this study as follows:

“In this study, by using a mediation model, we explored empirical support for the associations between changes in the different concepts in the direction described in the adapted conceptual framework on the working mechanisms of IMR.

The original RCT from which the data derived included three time points of measurement. However, to be able to empirically test the adapted IMR conceptual framework using a longitudinal model, for instance by using a random intercept cross-lagged panel model (56), required measurements at four time points. This is because the proposed working of IMR begins with a suggested change in illness self-management (M1-M2), which could cause a change in functional and clinical recovery (M2-M3), which in turn could cause a change in personal recovery (M3-M4). Therefore, by only using the data of the three available measurement points we were not able to execute a longitudinal analysis of the total adapted conceptual framework.” (lines 396-405)

**2. Second, the fact that the sample included participants assigned to both the Illness Management and Recovery and usual care conditions makes results interpretation difficult. The general conclusion seems to be that Illness Management and Recovery is indicated to promote personal recovery, but the analyses were not set up to support this claim (1). The researchers might consider limiting their sample to only those who participated in IMR (2), or, at the very least, entering treatment condition as a covariate into the mediation models (3).**

**Answer:**

Thank you for your comments and suggestions. Illness self-management concerns people’s capabilities to take care of themselves and regain control of their lives (5). This includes the ability to perform the tasks needed to manage and live successfully with the physical, social, and emotional consequences of a serious and persistent condition (3) (lines 56-59). The current analysis concerns an improvement in illness self-management skills (IM) measured with the IMRS client version. This concerns improvements in the illness self-management skills of *all* study participants (both the intervention as well as the control group) with measurements at both the first and third timepoints. Therefore, we were able to measure the association of IM improvement with the improvement of the other concepts over time. We consider the impact of illness self-management in this analysis as support for the relevance of IMR as IMR works to improve illness self-management **(1).** Moreover, we see the results of illness self-management in this analysis as support for the relevance of the adapted IMR theoretical model to better understand the associations between the variables in the model over time.

Reviewer # 1 suggests entering the treatment condition as a covariate into the mediation models to control for the treatment condition. We agree that this would be consistent with using participants of both experimental conditions in our analysis. Therefore, we executed the analysis again entering the treatment condition as a covariate into the mediation models **(3)**. The associations between the variables in the model changed only slightly. The conclusions of the analysis did not change.

Limiting our sample to only those who participated in IMR would statistically reduce the power of the analysis. A larger and more heterogeneous group offers more statistical power and more stable results. Limiting our sample does not seem necessary to determine the relationships between the concepts in the overall model, because the study participants in the control group also underwent changes in their illness self-management skills **(2)** (12).

In the statistical analysis section we added the following paragraph: “Limiting our sample to only those who participated in IMR did not appear necessary to determine the relationships between the concepts, because the study participants in the control group also underwent changes in their illness self-management skills (12). A larger and more heterogeneous group offers more statistical power and more stable results **(2).** Consistent with using participants of both experimental conditions in our analysis, we executed the analysis entering the treatment condition as a covariate into the mediation models **(3).”** (lines 236-241)

We added the following to the Discussion: “We consider the impact of illness self-management in this analysis as support for the relevance of IMR as IMR works to improve illness self-management” (lines 374-376).

Minor issues:

**3. Please state what conceptual framework is being used to support the study. CHIME is mentioned, but it is not clear that this is the framework on which the study is based.**

**Answer**: This regards the conceptual framework of the IMR program (4, 11,12). This framework is a theoretical model on the working mechanisms of IMR. It is relevant because the research question of this article is partly based on a variant of the framework we created. It is mentioned several times in this paper, starting with lines 104-107. We added the original version of the conceptual framework

of IMR as supplementary material to our article (S1 appendix A). (line 107)

**4. The authors might consider citing Thomas et al.’s (2018) meta-analysis of person-oriented recovery interventions, which found that interventions including IMR were associated with improvement in personal recovery.**

**Answer**: Thank you for your suggestion. Accordingly, we have cited Thomas et al.’s (2018**)** study in the Discussion section**:** “Additionally, our results are supported by a review and meta-analysis of person-oriented recovery interventions which emphasize psychoeducation, developing self-management skills, and fostering self-direction. This meta-analysis involved seven RCTs (N=1,739), including one on IMR, and indicated that these interventions can promote personal recovery (50).” (lines 319-323)

**5. Were participants screened for eligibility based on self-report?**

**Answer**: In designing the study our research team discussed this relevant subject. In the end we considered that practitioners are responsible for treatment. Therefore, the recruitment procedure for participation in the RCT was as follows: During interviews with an assistant researcher, the caseloads of all clinicians in the community mental health teams were reviewed by the clinicians themselves for their potential suitability for IMR. The clinicians asked the selected clients about their willingness to participate in IMR and to be informed of the study. If clients agreed, an assistant researcher explained the study objectives, the randomization procedure, and the three measurement moments. If written informed consent was given, the baseline interview was followed by randomization. In this way, written informed consent was obtained from all participants prior to participation(2). Participation in the study was not mandatory for participation in IMR.

In the section Study setting and data collection we reformulated the lines as follows: “In line with IRB regulations, participants for this RCT were recruited through clinician referrals for IMR. The selected clients indicated both a willingness to participate in IMR and to be informed about the study. An assistant researcher then explained the study objectives and procedures. If participants agreed to participate in the study, written informed consent was obtained (12). From Oct. 25, 2012, to May 2, 2014, 187 study participants were recruited. In this RCT, 116 participants (3:2 ratio) were randomly assigned to receive IMR + Care as Usual (n = 116) or Care as Usual alone (n = 71) (2). We calculated that this study size was appropriate for this RCT (12).” (lines 160-167)

**6.** **To enhance interpretability of study findings, descriptive statistics pertaining to the study measures at each time point should be provided.**

**Answer:** Thank you for your advice. Accordingly, at the end of the paragraph on mediation analysis, we added: “To enhance the interpretability of the study findings, descriptive statistics pertaining to the study measures at T1 and T3 of the mediation analysis are provided in Table 3.” Table 3 was added (lines 299-303).

| **TABLE 3:**  Mean (SD) scores on four concepts on T1 and T3 of the mediation analysis (N=165) | | |
| --- | --- | --- |
|  | Mean (SD) | |
|  | T1 | T3 |
| Illness Self-management (IMRS Client version) | 3.32 (0.49) | 3.49 (0.52) |
| Clinical Recovery (BSI) | 1.25 (0.83) | 1.16 (0.83) |
| Functional Recovery (SFS) | 105.49 (8.64) | 107.17 (9.05) |
| Personal Recovery (MHRM) | 70.55 (20.25) | 72.51 (19.56) |
| IMRS= Illness Management and Recovery scale. BSI = Brief Symptom Inventory. SFS= Social Functioning Scale | | |
| MHRM= Mental Health Recovery Measure. | | |

**7. Suggest reframing the results section to more directly answer the stated research question: 1) the association of changes over time in the illness self-management skills of people with SMI on changes in personal recovery, and 2) the degree to which this association is mediated by changes in clinical and functional recovery.**

**Answer:**

Thank you for your suggestions. Accordingly, we reformulated the section on mediation analysis as follows:

“From a multiple mediation analysis, it appears that improvements in the overall illness self-management skills of people with SMI directly affects improvements in overall personal recovery, independent of its impact on improvements in clinical and functional recovery (c' = 0.30).

It was also shown that the improvement in overall illness self-management indirectly affected improvements in overall personal recovery through its effect on the improvement of clinical and functional recovery. However, this indirect impact appeared to be less pronounced.

As shown in Fig 2 and Table 2, clients who improved more in overall illness management improved more in clinical and functional recovery than those clients who improved less in overall illness management, and clients who improved more in clinical or functional recovery also improved more in personal recovery. Both confidence intervals of the indirect pathways via improvement of clinical recovery (path *a_1_b_1_* = 0.50 x 0.27 = 0.13) and of the pathways via improvement of functional recovery (*a_2_b_2_* = 0.41 x 0.21 = 0.08) were above zero, implying statistical significance.

These results suggest that illness self-management appeared to be a stronger direct determinant of personal recovery than indirectly via clinical and functional recovery” (lines 274-288).

**8. I am not sure that functional recovery as a mediator between illness management and personal recovery reflects a variant of the IMR framework, as functional recovery is sometimes considered as synonymous with clinical recovery (see:** **Liberman et al. 2002).**

**Answer:** In the article, we indicate that indeed “Recovery is a complex and multi-dimensional concept that has been defined in several ways (7-10).”(line 69) We have added Liberman et al. 2002 to these references (10) (line 70)**.** Additionally, we provide the definitions used in this study that differentiate between clinical and functional recovery: “In a typology used in previous studies (4, 11, 12), which will be used in the present study, three types of recovery can be identified, which should be considered, not mutually exclusive, but complementary aspects of recovery (13). The first type is clinical recovery, which concerns the degree of psychiatric symptomatology (14-16). The second type is functional recovery which can be defined as the degree of vocational and social functioning, such as acting according to age-appropriate role expectations, the performance of daily living tasks without supervision, engagement in social interactions (17), and the degree of independence with regard to housing (9, 18)” (lines 70-76).

The original conceptual framework of the IMR (Mueser et al. 2006) defined the different types of recovery in a similar way to the variant of this IMR framework that we used in this mediation analysis. Therefore we added Mueser et al. 2006 as reference (4) at the beginning of the sentence: “In a typology used in previous studies (4,11,12) which was used in the present study, three types of recovery were identified, which should be considered as not mutually exclusive, but complementary aspects of recovery (13).” (lines 70-71).

Reviewer #2:

**Dear Author(s),
I commend your work on the association between illness self-management skills and personal recovery in SMI patients. Your methodological rigor, especially the bootstrap techniques and mediation analysis, is admirable.**

Thank you for your encouraging comments.

**1. Abstract: Consider moving the "clinical trial registration number" to the methods section if it doesn't add critical value in the abstract.**

**Answer:** Following your advice, we have removed the mention of the “clinician trial registration number” from the abstract. This registration number was already part of the methods section (line 186).

**Introduction**:
**2 Elaborate briefly on previous study methodologies and findings.**

**1. To strengthen the manuscript's foundation, a brief but comprehensive summary of the previous study's methodology and findings would be helpful. This can ensure that readers understand the context in which the previous conclusions were drawn. For each cited study or meta-analysis, it could be beneficial to provide a brief summary of their methods, sample sizes, and key findings to lend weight to the argument.**

**Answer:** Thank you for your insights. In the following paragraphs we quote the text from our article regarding previous research and then cite the text we have added to each paragraph.

In the **Introduction** we mention:

“Previously, in a review of illness management research for persons with severe mental illnesses, a close relationship between illness management and personal recovery was suggested because the improved management of symptoms, relapses, and stresses of everyday life is critical to developing hope and reaching personal recovery goals (5). This review was based on an empirical literature review on teaching strategies for illness self-management (5). It included 40 RCTs. Key findings were that the five aforementioned empirically supported illness self-management strategies were identified including psychoeducation, cognitive-behavioral interventions, developing a relapse prevention plan, and social and coping skills training (5)” (lines 80-87).

Within these lines we added: “This review was based on an empirical literature review on teaching strategies for illness self-management (5). It included 40 RCTs. Key findings were that the five aforementioned empirically supported illness self-management strategies were identified including psychoeducation, cognitive-behavioral interventions, developing a relapse prevention plan, and social and coping skills training (5)” (lines 83-87).

“The working of IMR is suggested in the theoretical conceptual framework for the Illness Management and Recovery program (4, 24). This conceptual framework suggests that better illness management leads to better clinical recovery, and further that better clinical recovery leads to more personal and functional recovery, (S1 Appendix A)” (lines 104-107).

In this paragraph we added the word ‘’theoretical’’ (line 105).

“Based on this conceptual framework, in a previous study, using structural equation modeling, we cross-sectionally analyzed the association of five components of illness management with the three types of recovery (4). This analysis used the baseline data of participants in our RCT on IMR (N = 187) (2, 4). The results showed that one illness management component—coping—was associated with clinical, functional, and personal recovery. The direct associations between coping and functional and personal recovery were stronger than the indirect associations via clinical recovery. One conclusion of this study was that clinical recovery appeared not to be a prerequisite for functional and personal recovery (4)” (lines 108-114).

Within this paragraph we added: “…. using structural equation modeling, …”(line 108). ”This analysis used the baseline data of participants in our RCT on IMR (N = 187) (11, 12)” (line 110).

“The current study was also conducted in the context of our RCT (N = 187) investigating the effects of IMR training in outpatients with SMI (12, 24). In this RCT multilevel modeling was used to investigate group differences over an 18-month period comprising 12 months of treatment and six months of follow-up. In this RCT we found a significant effect of IMR + care as usual versus care as usual alone in self-reported overall illness management, as well as in personal recovery over an 18 month period (12). Moreover, in this RCT, clinical and functional recovery over time significantly improved about as much in both the IMR training group as in the care as usual group (12)” (lines 115-121).

Within this paragraph we added the sample size: “(N = 187)” (line 115), and the sentence:

“In this RCT multilevel modeling was used to investigate group differences over an 18-month period, comprising 12 months of treatment and six months of follow-up” (lines 116-117).

Second, in an earlier review and meta-analysis, including 37 RCTs with 5790 participants, illness management appeared to enhance clinical, personal, as well as functional recovery (3).”

Within these lines we added: … “including 37 RCTs with 5790 participants,” and “…clinical, personal, as well as…” (lines 127-128).

Third, in two other earlier reviews and meta-analyses, functional recovery was associated with personal recovery (16, 25). One of these two reviews, based on eight studies (N = 1938) found a small but significant effect size (r=.21) (16). In the other review, including 46 studies and using a total of eight personal recovery measures, showed small positive correlations with social factors (support, work and housing, and functioning; r = 0.23-0.31) (25) (lines 129-133).

Within these lines we added: “One of these two reviews based on eight studies (N = 1938), found a small but significant effect size (r=.21) (16). The other review, including 46 studies using a total of eight personal recovery measures, showed small positive correlations with social factors (support, work and housing, and functioning; r = 0.23-0.31) (25)” (lines 130-133).

**3-Clarify the shift from prior cross-sectional studies to the current RCT.**

**Answer:** Thank you for this opportunity to expand on this. We shifted from our prior cross-sectional study using data from the baseline measurement of our prior RCT (Roosenschoon et al. 2019) to the current mediation analysis using data from all three moments of measurement of our prior RCT (Roosenschoon et al. 2021), because we aimed to explore the effect of change over time. This is mentioned in the following sentences:

“Based on these results, we wondered whether the found changes in illness self-management might be related to the found changes in personal recovery. If this were the case, we wondered whether this association could be (partially) mediated by changes in clinical and functional recovery” (lines 124-126).

**4-Expand on theoretical or empirical evidence that suggests potential mediating relationships.**

**Answer:** Thank you for this opportunity to bring this to you. In the next few lines, we explore theoretical and empirical evidence that suggests possible mediating relationships.” (lines 115-140).

**“**The current study was also conducted in the context of our RCT (N = 187), investigating the effects of IMR training in outpatients with SMI (12, 24). In this RCT multilevel modeling was used to investigate group differences over an 18-month period, comprising 12 months of treatment and six months of follow-up. In this RCT we found a significant effect of IMR + care as usual versus care as usual alone in self-reported overall illness management, as well as in personal recovery over an 18 month period (12). Moreover, in this RCT, clinical and functional recovery over time significantly improved about as much in both the IMR training group as in the care as usual group (12).

Based on these results, we wondered whether the found changes in illness self-management might be related to the found changes in personal recovery. If this were the case, we wondered whether this association could be (partially) mediated by changes in clinical and functional recovery. Rationales for applying these two mediators in the present analysis were: First, in the proposed working of IMR, clinical recovery was a mediator between illness management on the one hand and personal and functional recovery on the other (4). Second, in an earlier review and meta-analysis, including 37 RCTs with 5790 participants, illness management appeared to enhance clinical, personal as well as functional recovery (3). Third, in two other earlier reviews and meta-analyses functional recovery was associated with personal recovery (16, 25). One of these two reviews based on eight studies (N = 1938) found a small but significant effect size (r=.21) (16). In the other review, including 46 studies, using a total of eight personal recovery measures showed small positive correlations with social factors (support, work and housing, and functioning; r = 0.23-0.31) (25). Therefore, the research question arose as to whether functional recovery, as well as clinical recovery, might be a mediator that could explain the relationship between illness management and personal recovery.

Therefore, on the basis of the working of IMR suggested in the conceptual framework (4), the results of our RCT (12), and the results of three reviews and meta-analyses, we were interested to explore in this study to what degree changes over time in overall illness self-management might be associated with changes in personal recovery. Furthermore, we wanted to identify whether these associations might be (partly) mediated by changes in clinical or functional recovery” (lines 115-140).

In these lines empirical evidence is shown (1) in the results of our own RCT on IMR (12). In this RCT we found statistically significant improvement in illness self-management and statistically significant improvement in personal recovery, but not in functional recovery. (2) In addition, in this RCT we found a main effect of time in both clinical and functional recovery. (3) Moreover, we mention empirical evidence of an earlier review and meta-analysis on the effects of illness management on clinical, personal, and functional recovery (3). (4) Furthermore, in two other earlier reviews and meta-analyses empirical evidence was shown on the association of functional and personal recovery (16, 25). Theoretical evidence is shown in the conceptual framework on the proposed working of IMR, which suggests that improvement in illness self-management would lead to improvement in personal and functional recovery mediated by clinical recovery (4). These combined results led us to hypothesize that improvement in illness self-management would be a determinant of improvement in personal recovery, mediated by improvement in clinical and functional recovery.

**5. Address the discrepancy in the 3:2 ratio of IMR + CAU to CAU alone participants. Ensure clarity on randomization procedures and reasons for any deviations.**

**Answer**: For the allocation of participants to receive IMR plus CAU (i.e., the intervention group) or CAU only (i.e., the control group), we used computer generated random permuted blocks of five stratified across the treatment teams. Blocks were independently constructed by a research assistant using a randomization plan (<http://www.randomization.com>) (24) This means that a list of blocks of five was generated by the randomization website, in which the order of the experimental and control condition was randomly determined, under the condition that each block contained three times the "experimental condition" and twice the "control condition. Each block was used for one treatment team, as mentioned above. When a block was fully used, the next empty block was used for a new client included in the study. This block was then used only for subsequent study participants of this team only.

The results of our pilot study suggested an expected 50% dropout rate from treatment within the experimental condition (23). Therefore, to facilitate completer analyses, we planned to allocate more participants to the intervention group than to the control group with a ratio of 3:2 (24). The final ratio of numbers per experimental group reflects the result of the chosen randomization method: 116 participants for IMR + CAU and 71 participants for CAU alone.

Based on a suggestion from reviewer #1, in the analysis we applied the experimental condition as a co-variate. This means that in our mediation analysis we controlled for the condition. Therefore, for the mediation analysis the randomization ratio appeared irrelevant.

**6 a. Measures: Provide more details on the reliability, validity, and potential translation (English to Dutch) of the tools used.**

**Answer:**

In the section, Measures, we added multiple notes on the psychometric qualities of the four measures used in the analysis, including three Dutch translations of these measures.

The new text is as follows:

**Illness (self-) management**

We changed the text regarding the measure of Illness (self-) management as follows:

Illness (self-) management (IM) was assessed with the IMR scale client version. The IMR scale client version is a composite measure of various components of illness self-management. The 15 items rated on a scale of 1–5 comprise key IM elements, which are also trained in the IMR program, and include progress towards goals, knowledge regarding mental illness, relapse-prevention planning, involvement with significant others, coping with symptoms, medication adherence, substance abuse, and symptom distress (4, 24, 32-34). The IMR scales have good validity and moderate reliability (33, 35). To identify and correct discrepancies, the Dutch translation was independently back-translated into English and compared with the original version. Evidence has been provided for the reliability and validity of this Dutch version (24, 34).

For the IMR scale, three subscales were found: ‘Coping with Illness Outcome,’ ‘Knowledge and Goals,’ and ‘Effective Medication Use/Reduced Alcohol and Drug Abuse’ (35). The total score of the IMR scale client version was used for this analysis (lines 194-204).

We added: “The IMR scales have good validity and moderate reliability (33, 35). To identify and correct discrepancies, the Dutch translation has been independently back translated into English and compared with the original version. Evidence has been provided for the reliability and validity of this Dutch version (24, 34)” (lines 199 – 202).

We also added the following: ”The total score of the IMR scale client version was used for this analysis” (lines 203 – 204).

**Personal recovery**

We changed the text regarding the measure of personal recovery as follows:

“Personal recovery (PR) was assessed with the Mental-Health Recovery Measure (MHRM) (36). The MHRM, whose 30 items are self-rated on a scale of 0–4, is a composite personal recovery scale that measures self-empowerment, learning, new potentials, and spirituality. The authorized Dutch translation (37) is a reliable measure in terms of internal consistency; convergent and divergent validity are generally acceptable (37-39) (lines 206-210).”

We added: “The authorized Dutch translation (37) is a reliable measure in terms of internal consistency; convergent and divergent validity are generally acceptable (37-39)” (lines 208-210).

**Clinical recovery**

We changed the text regarding the measure of clinical recovery as follows:

“Clinical recovery (CR) was assessed with the Brief Symptom Inventory (BSI). The BSI is self-rated and has 53 items rated on a scale of 0–4. The authors report good validity, internal consistency, and test-retest reliability for the nine symptomatology dimensions: Psychoticism, Depression, Somatization, Phobic Anxiety, Obsessive Compulsive, Interpersonal Sensitivity, Anxiety, Hostility, and Paranoid Ideation. Good test-retest reliability for the three Global Indices is also reported: global severity index (GSI), positive symptom total (PST), and positive symptom distress index (PSDI) (39-41). These psychometric qualities also relate to the Dutch translation of the BSI (42, 43) (lines 212-218).

Within these lines we added: “The authors report good validity, internal consistency, and test-retest reliability for the nine symptomatology dimensions” (lines 214-215), and “also good test-retest reliability for the three Global Indices: global severity index (GSI), positive symptom total (PST), and positive symptom distress index (PSDI) (39-41). These psychometric qualities also relate to the Dutch translation of the BSI (42, 43)” (lines 213-218).

**Functional recovery**

We changed the text regarding the measure of functional recovery as follows:

“Functional recovery (FR) was assessed with the Social Functioning Scale (SF-scale). The SF-scale is a self-administered questionnaire that measures social and role functioning with 76 items with varying response formats on the following seven dimensions: social withdrawal, relationships, social activities, recreational activities, independence (competence), independence (performance), and employment. This scale has been described as reliable, valid, sensitive, and responsive to change (11, 39, 44, 45)” (lines 220-224).

Within these lines we added: “This scale has been described as reliable, valid, sensitive, and responsive to change (11, 39, 44, 45)” (lines 223-224).

**6 b.** **Elaborate on the IMR program's components, objectives, and methodologies, considering its centrality to the study.**

**Answer:**

We fully agree with the suggestion of the reviewer. We would like to take this opportunity and add information on objectives, components, and part of the methodologies of IMR in the following lines:

“Several illness self-management training programs have been developed including Illness Management and Recovery (IMR). The aim of IMR is to achieve progress in recovery by combining better illness management with the pursuit of personal goals (4). IMR is currently used in several countries such as the US, Europe, and Asia.

Illness self-management concerns people’s capabilities to take care of themselves and regain control of their lives (5). This includes the ability to perform the tasks needed to manage and live successfully with the physical, social, and emotional consequences of a serious and persistent condition (3). More specifically for people with SMI, it includes the ability to reduce their susceptibility to the illness, and to effectively cope with their symptoms. For example, it is necessary to have knowledge of mental illness to be able to make informed treatment decisions along with professionals, and to have the ability to reach out for social support (5, 6).

Complementary to this, IMR, a structured psychosocial intervention supporting illness self-management, includes providing psycho-education and teaching skills for informed decision-making on treatment; providing cognitive-behavioral training to support behavioral tailored medication adherence; teaching coping skills to deal with symptoms or stress; developing a relapse prevention plan; and improving social support through social skills training (3, 4)” (Lines 52-67).

Additionally:

“The aim of IMR training is to improve illness management skills and thereby improve clinical recovery which improves personal and functional recovery. This working of IMR is suggested in the theoretical conceptual framework for the Illness Management and Recovery program (4, 24). This conceptual framework suggests that better illness management leads to better clinical recovery, and that better clinical recovery leads to more personal and functional recovery (S1 Appendix A)” (lines 103-107).

We added: “In practice IMR is a comprehensive structured training program for people with SMI. In our RCT IMR was provided in a group format with up to eight people in weekly 90-min sessions for an average of 12 months (12). The IMR intervention comprised eleven modules as well as practitioner guides and handouts for the participants. The eleven modules included Recovery Strategies, Basic Facts about Mental Illness, the Stress-Vulnerability Model, Building Social Support, Using Medication Effectively, Drug and Alcohol Use, Reducing Relapses, Coping with Stress, Coping with Persistent Symptoms, Getting Your Needs Met in the Mental-Health System, and Healthy Lifestyles (22). Each group of patients who received IMR was guided by two trainers who used a combination of motivation enhancement strategies, educational strategies, and cognitive-behavioral techniques (4). During the first module—Recovery Strategies—the participants identified their personal goals during the program. For half of each session, some participants worked toward these goals. During the other half of each session, all participants worked on the module subjects with the assistance of handouts (12). Our pilot study demonstrated that completing the modules required an average of three to four sessions (23)” (lines 89-102).

**7. Results: Discuss the choice and implications of using difference scores versus raw post-intervention scores.**

**Answer:** Using raw post-intervention scores would require another cross-sectional analysis. The implications of this choice would be that it would no longer be possible to include the effect of change over time in the analysis.

In the Discussion paragraph we added the sentence: “To include the effect of change over time in the analysis, we chose difference scores over raw post-intervention scores.” (lines 370-371).

**Discussion:
8-Provide more supporting evidence for strong claims.**

**These results suggest that improvement in clinical and functional recovery might not be a prerequisite for improvement in personal recovery," is strong. To solidify this claim, you could provide more supporting evidence from the study and cite any corroborative external studies.**

**Answer:**

Indeed, our conclusion needs additional argumentation. The main result of the present mediation analysis is that **“**improvement in the overall illness self-management skills of people with SMI strongly directly affects the improvement in overall personal recovery independent of its impact on improvement in clinical and functional recovery” (lines 275-277). “These results suggest that illness self-management appears to be a stronger direct determinant of personal recovery than indirectly via clinical and functional recovery” (lines 287-288). “These results also suggest that improvements in clinical and functional recovery might not be a prerequisite for improvements in personal recovery” (lines 313-315).

We added: “ The conclusion that improvement in clinical and functional recovery may not be a prerequisite for improvement in personal recovery is supported by the following previous studies: First, our cross-sectional study, as mentioned in the introduction, showed that for predicting personal recovery, the ability to cope with symptoms (i.e. self-management) is more relevant than the symptoms themselves (11). Second, in our earlier RCT (N = 187) on the effects of IMR using multilevel modeling, we found a statistically significant effect of IMR + care as usual versus care as usual alone in self-reported overall illness management, as well as in personal recovery over an 18 month period (12). However, in this RCT, we found no statistically significant effects in clinical and functional recovery (12). Third, two previous meta-analyses on people with psychotic disorders indicated a small to moderate association between clinical and personal recovery, and showed that personal recovery was explained only partly by symptom severity (16, 25). The authors of one of these meta-analyses indicated that their findings imply that patients who still experience symptoms might nevertheless report good personal recovery. The same meta-analysis concluded that treatment and outcome monitoring of patients with schizophrenia spectrum disorders should pay attention separately to clinical and personal recovery (16). Fourth, a previous systematic review and meta-analysis (N=845) showed the efficacy of the Wellness Recovery Action Plan (WRAP) for improving personal recovery without improving clinical recovery outcomes (27). Fifth, several authors affirm the relevance of improvement in personal recovery and emphasize the importance of the therapeutic benefits of managing mental illness, contributing to personal well-being and self-perceived growth, as well as the value of leading a fulfilling life despite the presence of persistent symptoms (8, 9, 13, 19). For instance, over and above symptoms (8), the loss of self-esteem is a recognized component of the major impact of schizophrenia on an individual's life and self-image (53-55). The concept of self-esteem corresponds to the concept of identity (i.e., a positive sense of self), which is a relevant constituent of personal recovery (12, 21)” (Lines 336-357).

**9**-**Explore why clinical and functional recovery had 'weak' mediating effects.**

**Answer:** Kindly refer to the comprehensive supportive evidence cited in the answer to question 8, that improvement in clinical and functional recovery might not be a prerequisite for improvement in personal recovery. Therefore, we suggest that compared to the impact of illness self-management, the relatively 'weak' mediating effects of clinical and functional recovery are confirmed. The effects may be confirmed because the measures used for clinical and functional recovery, the BSI and the SF-scale respectively, might be considered robust instruments.

**10-Discuss similarities or differences with prior studies more explicitly.**

**Answer**:

See our answers to question 8 which elaborate on several prior studies that align with the current results.

**11-**Elaborate on how the results specifically support the relevance of IMR.

**Answer:**
We view the impact of illness self-management in this analysis as support for the relevance of IMR as IMR works to improve illness self-management.

We changed lines 378-381 as follows: “Given that in this study, improvements in self-management predicted improvements in personal recovery more directly than indirectly, we suggest that it can be considered supportive of the relevance of self-management interventions such as IMR. Therefore, in agreement with Lean et al., we suggest that these interventions should become part of the standard care for people with SMI and be given greater priority in guidelines (3).”

**12**-Address potential contradictions, e.g., the role of symptom management in developing hope for the future.

**Answer:**
Although some people might view this as a potential contradiction, we believe that “… for predicting personal recovery, the ability to cope with symptoms (i.e., self-management) is more relevant than the symptoms themselves (11)” (lines 338-339). In this sentence for clarity, we added: “(i.e. self-management)” (line 339).

Reviewer #3: The authors present results of a mediation analysis of data from a clinical trial in individuals with severe mental illness. In particular, they assessed whether changes in clinical recovery and functional recovery were mediators of the association between change in illness self-management (IM) and change in personal recovery. There mediation model is informed by theory, prior results and meta-analyses, so has justification. The authors identified both direct and indirect effects of IM on personal recovery, though the direct effect was much stronger. The manuscript is well written. I have some minor comments that would provide further clarification.

**1. In lines 30 and 201, authors state that difference scores are from subtracting means at baseline from means at the final time point for the change measures. Aren't these computed at the individual level? It is not clear what the "means" would be for each person. Certainly, at the group level, the average difference score would be the difference in means, but not at the individual level. Authors should clarify what is done.**

**Answer**: Thank you for your comments. We apologize for the ambiguity in this sentence. Difference scores were determined at the individual level. We changed this sentence to read: “Difference scores were constructed for all concepts by subtracting scores measured at T1 from scores at T3 (47)” Lines 28-29 and 233-234.

**2. In lines 175-176, authors indicate that IM has 3 subscales. It appears as though authors just use a single measure (at 2 time points) for IM - is this a total score? If so, authors should state that even though the scale has subscales, a total score was used for analysis (or if subscales were used, that should be clarified).**

**Answer**: At the end of this paragraph, we added the sentence: “However, the total score of the IMR

scale client version was used for this analysis” Lines 203-204.

**3. In line 216, authors refer to a regression equation from delta IM to delta PR as the direct effect. That is the path, but the regression equation actually includes the mediators, right?**

**Answer**: Indeed, the mediators are part of the regression equation. However, we suggest that the regression analysis with ΔPR as the outcome contains only c' and the b-paths. The a-paths are separate regression analyses with ΔIM as the predictor and the mediator as the outcome. Therefore, we used three regression analyses: one for a1, one for a2, and one for b1, b2 and c'.

We changed this sentence as follows: “The direct path is the effect of ΔIM on ΔPR (c’) from a regression analysis with the mediators as covariates (via *b1* and *b2*). The indirect paths were a combination of parameter estimates from regression analyses with ΔIM as a predictor and each of the mediators as outcome (via *a1* and *a2*) and the parameter estimates regarding the covariates in the aforementioned regression analysis (Fig 1)” Lines 255-259.
