# Supplementary material for: Illness (self) management, clinical and functional recovery as determinants of personal recovery in people with severe mental illnesses: A mediation analysis
Source: PLoS One. 2024 Nov 26;19(11):e0313202. doi: 10.1371/journal.pone.0313202 (PMC11594398; doi:10.1371/journal.pone.0313202)
Supplement: Attachment — Submitted filename: Reviewer 2 recommendation (Revise version).docx [file pone.0313202.s007.docx]

**Reviewer 2 recommendation (Revise version)**

**Illness (self) management, clinical and functional recovery as determinants of personal recovery in people with severe mental illnesses: A mediation analysis**

1. I suggested moving the clinical trial registration number to the methods section, and this recommendation was followed in the revised manuscript.

2. I asked for more elaboration on the transition from cross-sectional studies to the current RCT and details on the mediating relationships. These suggestions were addressed, as the revised version provides more clarity.

3. The issue of a 3:2 randomization ratio was raised. The revised version briefly addresses this but does not go into great depth about the reasons behind the deviation from randomization.

4. I asked for more details about the validity and reliability of the tools used. This was addressed more thoroughly in the revised version, including an additional explanation of the measures.

5. The revised manuscript still uses different scores despite reviewer concerns. The authors explained their rationale but did not change this methodological choice.

6. I suggested strengthening the discussion of the weak mediating effects of clinical and functional recovery. This was partially addressed in the revised manuscript.
